# Computational screening of medicinal plant phytochemicals to discover potent pan-serotype inhibitors against dengue virus

**DOI:** 10.1038/s41598-018-38450-1

**Published:** 2019-02-05

**Authors:** Muhammad Tahir ul Qamar, Arooma Maryam, Iqra Muneer, Feng Xing, Usman Ali Ashfaq, Faheem Ahmed Khan, Farooq Anwar, Mohammed H. Geesi, Rana Rehan Khalid, Sadaf Abdul Rauf, Abdul Rauf Siddiqi

**Affiliations:** 1 College of Informatics, Huazhong Agricultural University, Wuhan, P.R. China; 2 Department of Biosciences, COMSATS University Islamabad, Islamabad, Pakistan; 3 School of Life Sciences, University of Science and Technology of China, Hefei, P.R. China; 4 Department of Bioinformatics and Biotechnology, Government College University Faisalabad, Faisalabad, Pakistan; 5 Key Laboratory of Agricultural Animal Genetics, Breeding and Reproduction, Ministry of Education China, Huazhong Agricultural University, Wuhan, P.R. China; 6 Department of Chemistry, University of Sargodha, Sargodha, Pakistan; 7 Department of Chemistry, College of Sciences and Humanities, Prince Sattam Bin Abdulaziz University, Al Kharj, Saudi Arabia; 8 Department of Computer Science, Fatima Jinnah Women University, Rawalpindi, Pakistan

## Abstract

Emergence of Dengue as one of the deadliest viral diseases prompts the need for development of effective therapeutic agents. Dengue virus (DV) exists in four different serotypes and infection caused by one serotype predisposes its host to another DV serotype heterotypic re-infection. We undertook virtual ligand screening (VLS) to filter compounds against DV that may inhibit inclusively all of its serotypes. Conserved non-structural DV protein targets such as NS1, NS3/NS2B and NS5, which play crucial role in viral replication, infection cycle and host interaction, were selected for screening of vital antiviral drug leads. A dataset of plant based natural antiviral derivatives was developed. Molecular docking was performed to estimate the spatial affinity of target compounds for the active sites of DV’s NS1, NS3/NS2B and NS5 proteins. The drug likeliness of the screened compounds was followed by ADMET analysis whereas the binding behaviors were further elucidated through molecular dynamics (MD) simulation experiments. VLS screened three potential compounds including Canthin-6-one 9-O-beta-glucopyranoside, Kushenol W and Kushenol K which exhibited optimal binding with all the three conserved DV proteins. This study brings forth novel scaffolds against DV serotypes to serve as lead molecules for further optimization and drug development against all DV serotypes with equal effect against multiple disease causing DV proteins. We therefore anticipate that the insights given in the current study could be regarded valuable towards exploration and development of a broad-spectrum natural anti-dengue therapy.

## Introduction

By the last few years, dengue fever remains a constant threat in the tropical and subtropical regions worldwide. World Health Organization (WHO) estimates 100 million cases of dengue fever per annum. Of these, 500,000 cases require hospitalization, and in 25,000 cases conditions become worst which may lead to death. A recent study reported 390 million dengue infections worldwide per year; an infection toll more than three times the numbers given by World Health Organization (WHO)^1^. Despite of significant research developments, the medical science is still unable to deal with the antigenic variations among dengue serotypes as no specific drug has yet been launched in the market for this disease.

Dengue virus (DV) has been classified as member of *Flaviviradae* family. Members of this family cause multiple infections in humans such as dengue fever, tick-borne encephalitis, West-Nile fever and yellow fever. Four well-studied globally known serotypes including DV-1, DV-2, DV-3 and DV-4 exist which exhibit more than 70% primary sequence homology, and significant GC% conservation. Therefore, disease caused by all these serotypes share common symptoms^2^. Infection due to one DV serotype will confer lasting homotypic immunity but imparts immune-pathological responses in patients which predispose them to other DV heterotypic re-infection. Sequential infections by multiple DV serotypes result in more severe disorders such as organ impairment and bleeding etc. Dengue hemorrhagic fever (DHF) and dengue shock syndrome (DSS) typically occur through antibody-mediated disease enhancement (ADE), either from previous DV infection or from vaccine-induced ADE^3^. Despite having less sequence level variations, all these serotypes respond differentially against drugs. Presence of multiple serotypes of DV has hampered the efforts to develop effective drugs or vaccines against DV^4^. Additionally, dengue specific complexities linked to immune enhancement make it an extremely challenging task to design effective and broad spectrum anti-dengue therapeutic solutions^5^.

**Figure 1:**
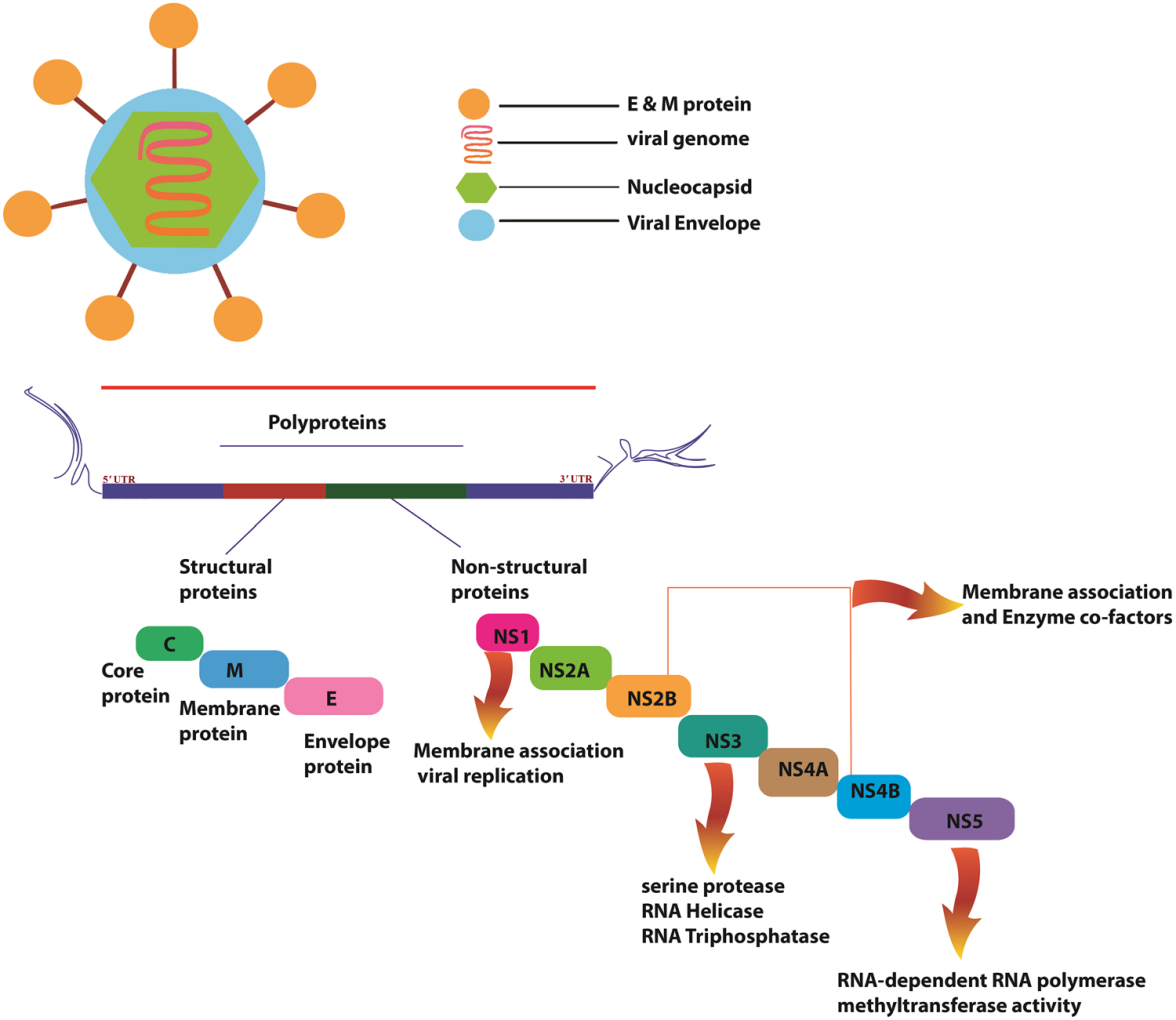
Diagram of Dengue virus RNA genome encoding three structural proteins namely core protein (C), membrane associated protein (prM, M) and envelope protein (E) and seven nonstructural proteins (NS1, NS2A, NS2B, NS3, NS4A, NS4B, and NS5).

These serotypes show antigenic variations in their envelope protein. In general, DV is characterized as a plus-strand RNA virus with 10.7 kb single strand RNA and approximately 50 nm viral envelope. Single strand RNA is translated into a single polyprotein chain followed by co-translational cleavage into 10 mature proteins^2^. These 10 mature proteins consist of three structural proteins (capsid (c), pre-membrane (prM), envelope (E)) and seven nonstructural proteins (NS1, NS2A, NS2B, NS3, NS4A, NS4B, and NS5) outlined in Fig. 1. Nonstructural proteins play major role in evasion of innate immune responses, virion assembly, and genome replication. Especially NS1, NS3 and NS5 are crucial for the formation of the viral particle during infection cycle^6^.

Nonstructural DV protein NS1, a highly conserved intracellular protein crucially involved in viral replication due to its two N-linked glycosylation sites (Asn-130 and Asn-207) which are utilized for addition of oligosaccharides during viral replication, and a potential biomarker is expressed on the surface of infected cell^7–9^. Crystal structure of NS1 reports three structural domains with distinct functions. Among these, α/β Wing and β-ladder domains are indispensable for viral replication within host cell as they mediate interaction with host’s intracellular membranous organelles. In NS1, twelve invariant cysteine residues that are involved in inter-domain interaction through disulfide bonds and three highly conserved glycosylation sites (Asn130, Asn175 and Asn207) are known to be important for its structural integrity and stability^8^. Various *in-vivo* and *in-vitro* studies are evident that Asn130 is crucial for viral growth, interaction with complement proteins, NS1 secretion, and cytopathic effect in cells while its loss results in compromised and attenuated DV^9^.

NS3 protease is the second largest non-structural DV protein which acts as a double-edged sword with its protease, helicase and/or RNA tri-phosphatase activity. NS3 crystal structure reports a classic chemo-trypsin-like fold with two β-barrels at its N-terminal and helicase and RNA tri-phosphatase domains at C-terminal. At the cleft between the two β-barrels, a highly conserved catalytic triad (His51-Asp75-Ser135) is of prime functional importance. For optimal enzymatic activity, β-barrel region of NS3 protease makes chimera with the hydrophilic part of NS2B (49–95 aa) which act like a cofactor by shielding hydrophobic residues of NS3^10^ Any disruption in functional activity of NS3/NS2B complex region results into the inhibition of viral replication and infectivity. Hence, to screen and evaluate effects of different drug candidates, NS3/NS2B complex has been considered a promising target protein^11–13^.

Likewise, the nonstructural DV protein NS5 has also been exploited as an attractive target for computational drug screening because of its dual enzymatic activity. Its N-terminus is comprised of a methyl-transferase (MTase) domain which protects nascent viral mRNA from degradation through RNA capping (post transcriptional modification), while RNA-dependent RNA polymerase (RdRp) domain located at its C-terminus is responsible for replication of positive strand RNA within the host cell^14–16^. Crystal structure of NS5 MTase with SAH and a nucleoside analogue, ribavirin triphosphate (RTP) reveals two binding sites: a *S*-adenosyl-_L_-methionine (SAM) binding site, which is highly positive charged and thus could serve as an RNA binding site during cap methylations, and lies in the same site for SAH; and an RNA cap site, which is also a GTP and GTP analogues-binding site^17^. Owing to its important biological function NS5 MTase/RdRp represents an ideal target for dengue virus therapy.

In contrast to conventional methods of drug screening which involves High Throughput Screening (HTS), recently Virtual High Throughput Screening (vHTS) term has been coined to accelerate the drug discovery for time-efficient identification of cost effective novel and selective drug leads. Conversely, HTS identified bulky hydrophobic drug candidates poorly suited to chemical amendments incurring higher costs and time^18^. Though, few vHTS success stories have been reported, identifying phytochemicals against individual DV proteins, none of them have sought to screen the compound bank of plant derivatives for concurrent inhibition of multiple conserved DV proteins of all known DV serotypes^19,20^. Phytochemicals are secondary metabolites and active ingredients of medicinal plants. These efficient biomolecules not only constitute defensive mechanism in plants but also control and lessen the severity of infections^21,22^. They provide their remedial function by foraging, scavenging and obstructing viral entry and DNA\RNA replication against a broad range of viruses. Recently, phytochemical based inhibitors and vaccine candidates against specific nonstructural DV proteins have been reported but none of them has been proved effective against dengue heterotypic infections^12,19,23–27^.

To discover a pan-serotype inhibitor, our idea was to target functionally and structurally conserved proteins of DV serotypes. High level of functional similarity between NS3 protease of all four serotypes have been reported previously^26^. While, DV2 and DV4 genome were reported be closely related with 70% sequence similarity^28^. Serological and molecular analysis revealed approximately 70% over all protein sequence identity between all the four distinct DV serotypes^29^. Moreover, comparable conserved GC% among DV serotypes genomes has also been reported previously. The percentage of GC contents in DV serotypes is associated to antigenic and genetic diversity, base composition, genome polarity, synonymous codon usage, and phylogenetic relationship of each serotype^30,31^. With all these ground-breaking details drug discovery approaches are underway to successfully inhibit conserved DV hotspots^10,23^.

This study aims at screening a set of compounds which could inhibit multiple serotypes of DV exploiting their conserved proteases. The study undertakes virtual ligand screening (VLS) to bring forth a set of phytochemicals capable to inhibit highly conserved non-structural DV proteins and also impede entire spectrum of DV serotypes promiscuously thus implementing a multi-target approach. To meet that goal, the study focused on targeting nonstructural DV proteins NS1, NS3/NS2B and NS5 which are responsible for viral replication and DV ADE phenomenon. A set of 10,326 phytochemicals were virtually screened against the aforementioned NS proteins of DV through structure based drug design approach and molecular dynamics (MD) simulations.

## Results

### Sequence and Structural Alignment Analysis

**Figure 2:**
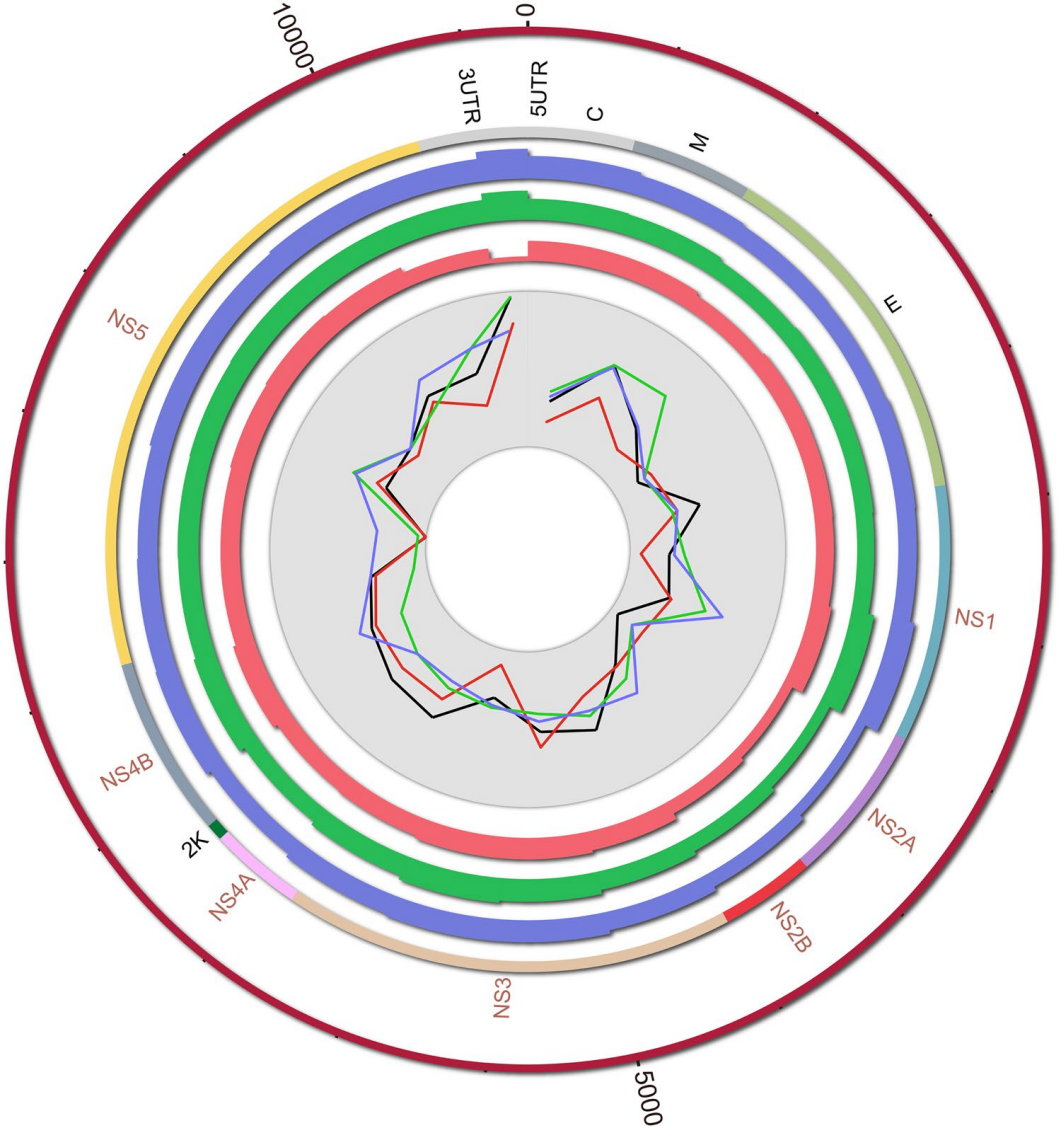
Comparison of all dengue virus serotypes’ whole genome sequences. Similarity and GC contents are calculated in 500 bp window size. Along centripetal axes the circles represent DV2 (reference), gene/protein regions of DV1, DV3 and DV4 are shown in blue, green and red‚ respectively. GC contents are represented with curvy lines in the inner most circle according to the aforementioned color scheme except the DV2, which is shown in black. Peaks of curves show the level of GC%age.

Comparative genome analysis of four DV serotypes identified numerous hubs of conserved regions and GC percentage as shown in Fig. 2. The outermost rim represents DV2 genome, rim next to it represents structural and non-structural proteins encoding regions and three subsequent circular plots in blue, green and pink represent DV1, DV3 and DV4 serotype genome‚ respectively. Circos plot highlights broad hubs on three inner rims which clearly demonstrate high sequence conservation within confined regions of genomes that specifically correspond to NS1, NS3 and NS5 protease of the four DV serotypes. GC percentage of four DV serotypes, shown as inward to outward curvy lines (blue for DV1; black for DV2; green for DV3 and red for DV4), varies from 42% to 54%. The curves show the presence of GC, base of curves shows low level of GC%, while peaks of curves represent the level of GC percentage (%age). Circos plot highlights distinct peaks of curvy lines in the inner most circle, which clearly demonstrates high level of GC% and conservation within confined and conserved sequence regions of DV serotypes genomes, specifically corresponding to NS1, NS3 and NS5. In our results DV-2 showed the highest GC %age. It is also noteworthy that 5′UTR region of all DV serotype genomes (DV 1–4) has low‚ while 3′UTR region has the highest level of GC %age. All information was calculated using 500 bp window (Fig. 2).

The sequence alignment of all for DV serotypes showed that identity between the set of four sequences of NS1, NS3 and NS5 proteins from each of the four DV serotypes (DV1-DV4) was found to be 73%, 79% and 77%‚ respectively. The sequence alignment also revealed that conserved residues Asn130 of NS1 (Supplementary Fig. 1), His51, Asp75 and Ser135 of NS3/NS2B (Supplementary Fig. 2) and Ser56, Gly83, Thr104, His110, Glu111, Asp131, Val132, Asp146 and Lys180 of NS5 (Supplementary Fig. 3), are also conserved among all 4 serotypes of DV.

Furthermore, the structural alignment of all four serotypes’ NS1 3D structures revealed conserved residue Asn130, inlaid at exactly same position in the binding pocket with an average RMSD nearly 0.15 Å as shown in Supplementary Fig. 4A. Similarly, aligned protein structures of NS3/NS2B chimera from four serotypes exhibits three conserved residues (His51, Asp75, Ser135) lying at similar positions within the same binding pocket with an average RMSD of 0.2 Å (Supplementary Fig. 4B). Likewise, analysis of superposed results of NS5 structures of four DV serotype demonstrates presence of conserved residues (Ser56, Gly81, Cys82, Arg84, Gly85, Thr104, Lys105, His110, Asp131, Val132, Asp146 and Gly148) in laid at same positions in binding pocket with an average RMSD of 0.753 Å (Supplementary Fig. 4C). Sequence and structural alignment clearly demonstrate well conserved functional residues within the active pockets of NS1, NS3/NS2B and NS5 among four DV serotypes.

### Database Screening and Molecular Docking

**Figure 3:**
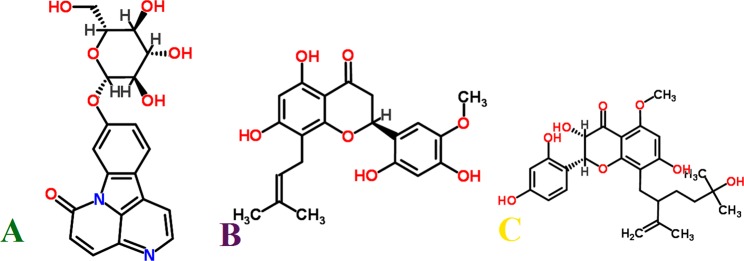
2D chemical structures of three top ranked multiple DV serotypes inhibitors. (**A**) Canthin-6-one 9-O-beta-glucopyranoside, (**B**) Kushenol W and (**C**): Kushenol K.

**Figure 4:**
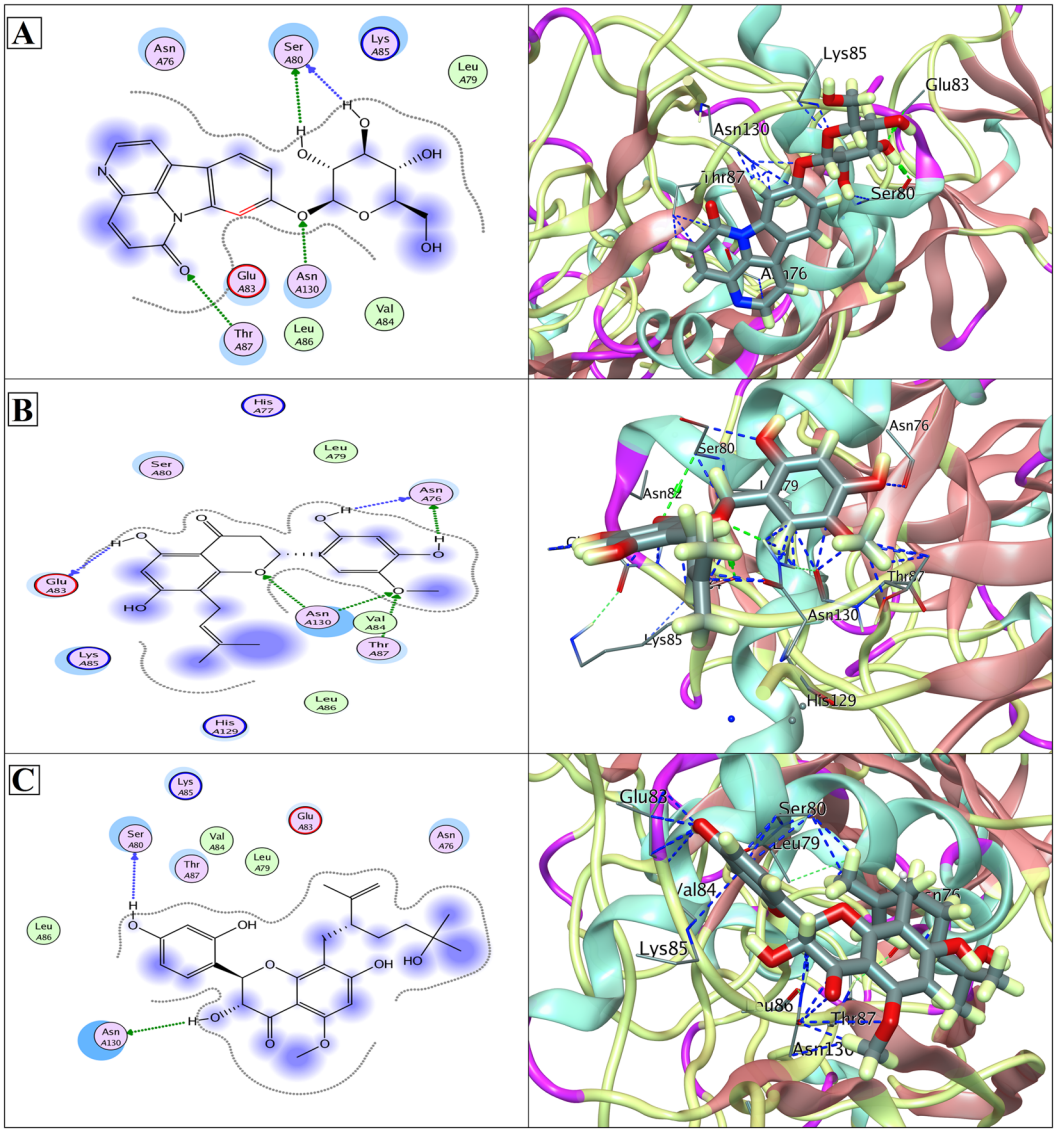
(**A**) Docked Canthin-6-one 9-O-beta-glucopyranoside in complex with NS1 protease (PDB ID: 4O6B); showing hydrogen bonds with the side chain/back-bone of SER80, and to side chains of THR87 and ASN130. (**B**) Docked Kushenol W in complex with NS1 protease; showing hydrogen bonds to the side chain/back-bone of ASN76, to the back-bone of GLU83, and to side chains of THR87 and ASN130. (**C**) Docked Kushenol K in complex with NS1 protease (PDB ID: 4O6B); showing hydrogen bonds to the back-bone of SER80 and to the side chain of ASN130. LigX 2D interaction analysis shown at left and 3D interaction analysis shown at right side.

Phytochemical ligand database was docked against the DV proteases and docked compounds were ranked based on a stringent filter which included four factors, maximum occupancy of binding pocket with minimum Gibbs free energy, strength of hydrogen bonding and other potential non-covalent interaction cumulatively estimated and represented with a S-score function. Out of 10,326 docked molecules, top ranking docking poses were selected. The ranking criteria involved criteria based on a set of thresholds, which required that a ligand should show the desired S-score values (lower the score stronger the interaction and affinities) and bind with all the selected NS protein target serotypes involving all the hotspot conserved residues of the binding pocket. Three phytochemicals including Canthin-6-one 9-O-beta-glucopyranoside, Kushenol W and Kushenol K (Fig. 3) were observed to bind with strong binding affinity within the active site of the selected DV proteins (Table 1). Canthin-6-one 9-O-beta-glucopyranoside was bound to NS1 with a score of −12.14 (Table 1A), forming hydrogen bonds with side chain/back-bone of SER80, and with side chains of THR87 and ASN130 of NSI, while other close interacting residues were Asn76, Leu79, Glu83, Val84, Lys85 and Leu86 Fig. 4A. Canthin-6-one 9-O-beta-glucopyranoside was followed by Kushenol W and Kushenol K with binding score of −11.89 and −10.91, Fig. 4B,C. Canthin-6-one 9-O-beta-glucopyranoside showed strong binding with NS1 active site residues followed by Kushenol W and Kushenol K whose binding score was marginally lower than Canthin-6-one 9-O-beta-glucopyranoside but their binding energies for NS1 active site were much higher. All the three ligands showed strong hydrogen binding with the conserved Asn130 and non-covalent interactions with Lys 85, Table 1A.

**Table 1:** Summary of top ranked phytochemicals screened against NS1, NS3/NS2B Chimera, NS5 RNA and NS5 SAM Pocket with their respective dock score, binding affinity, interacting and closer contact residues.

Phytochemical Name	Score	Binding Affinity (Kcal/mol)	Residues interacting with Ligand through H-Bonding	Closer Contact Residues
**A. NS1**				
Canthin-6-one 9-O-beta-glucopyranoside	−12.14	−15.08	**Asn130**, Ser80, Thr87	Lys85, Asn76, Glu83, Leu79, Val84, Leu86
Kushenol W	−11.89	−19.95	**Asn130**, Asn76, Thr87, Glu83	Lys85, His129, Ser80, Val84, Leu86, His77, Leu79
Kushenol K	−10.91	−17.05	**Asn130**, Ser80	Asn76, Thr87, Lys85, Glu83, Val84, Leu79, leu86
Reference (N-Acetyl-D-Glucosamine)	−8.15	−10.27	**Asn130**, Ser80	Lys85, Leu86, Thr87
**B. NS3/NS2B**				
Canthin-6-one 9-O-beta-glucopyranoside	−12.26	−15.17	**His51, Asp75, Ser135**, Gly151, Gly153, Asn152	Leu128, Tyr150
Kushenol W	−11.68	−14.55	**His51**, **Ser135**, Gly151, Gly153	**Asp75**, Tyr161, Leu128, Asn152, Pro132, Phe130
Kushenol K	−11.30	−16.39	**His51**, **Ser135**, Pro132, Tyr150	**Asp75**, Gly153, Leu128, Gly151, Asn152, Phe130
Reference(Glycerol)	−6.71	−9.33	**His51, Asp75**, Gly153	**Ser135**, Gly151, Asn152
**C. NS5**				
C1. RNA Pocket				
Canthin-6-one 9-O-beta-glucopyranoside	−15.72	−18.89	Gly83, Thr104, Glu111, Val132, Asp146, Ile147	His110, Phe133, Asp131, Gly81, Lys105, Glu149
Kushenol W	−14.55	−25.37	Gly81, Asp146, Lys180	Glu149, Ile147, Thr104, Tyr103, Ser56, Arg84, His110, Glu111
Kushenol K	−13.50	−22.79	Ser56, Gly109, Gly148	Lys180, Glu216, Ser150, Glu149, Lys61, Arg84, Glu111
Reference (S-Adenosylmethionine)	−12.31	−20.77	Val132, Asp146, Ser150, Lys180, Gly81	Arg211, Glu216, Cys82
C2. SAM Pocket				
Canthin-6-one 9-O-beta-glucopyranoside	−14.53	−26.31	Lys105, Asp131, Val132	Lys130, His110, Ile147, Glu149, Ap146, Gly148
Kushenol W	−13.91	−19.99	Thr104, Asp146	Ile147, Gly148, Gly83, Lys105
Kushenol K	−13.68	−32.24	Cys82, Arg84, Gly85, Asp146, Lys180	His110, Gly83, Lys105, Gly216, Glu149
Reference (S-Adenosylmethionine)	−12.21	−20.91	Lys105, Thr104, Glu149	Asp181, Arg180, His110, Gly83

**Figure 5:**
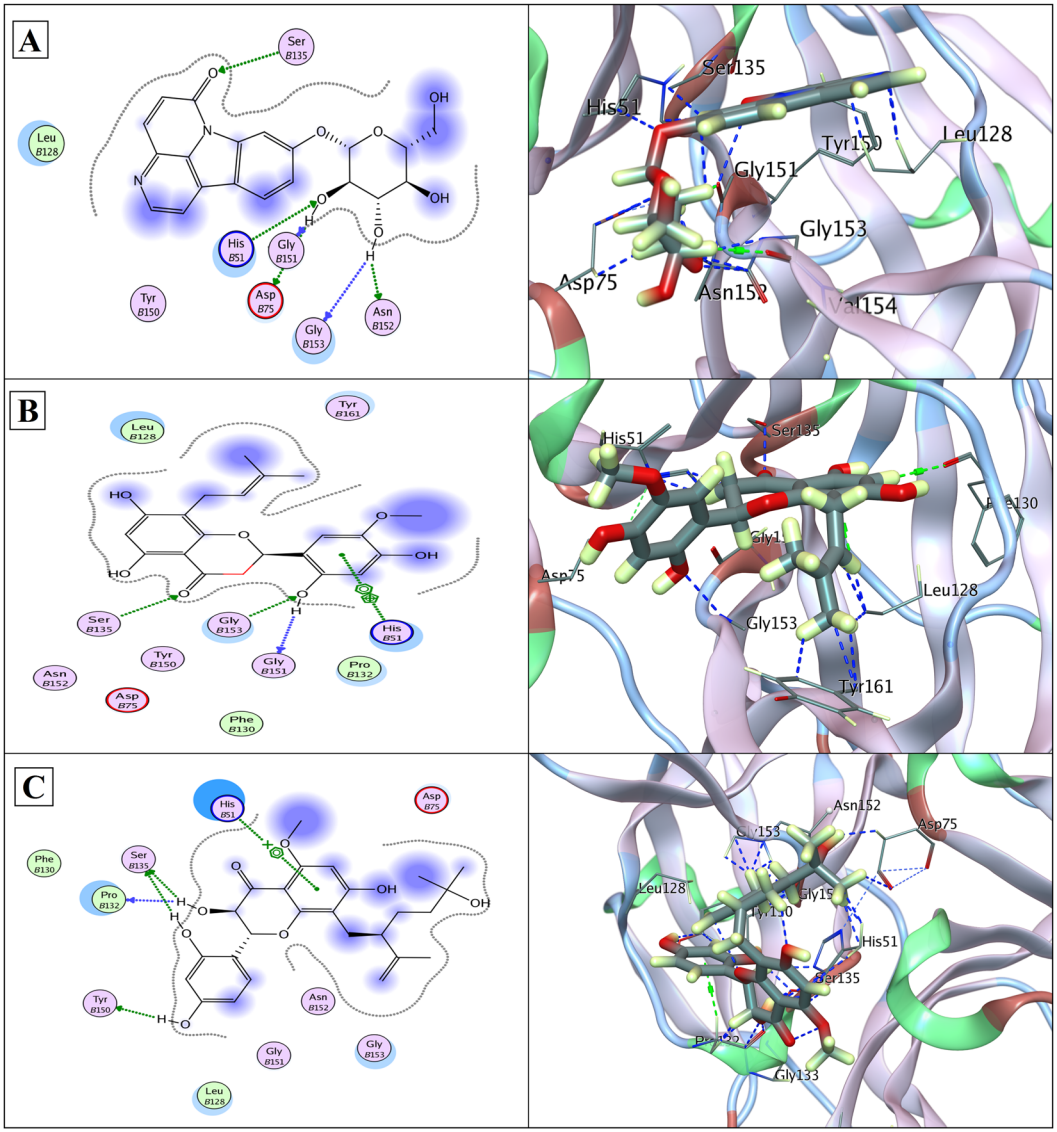
(**A**) Docked Canthin-6-one 9-O-beta-glucopyranoside in complex with NS3/NS2 protease (PDB ID: 2FOM); showing hydrogen bonds to side chains of HIS51, ASP75, SER135 and ASN152, and to the bank-bone of GLY151 and GLY153. (**B**) Docked Kushenol W in complex with NS3/NS2 protease (PDB ID: 2FOM); showing hydrogen bonds to side chains of SER135 and GLY153 and to the back-bone of GLY151. An arene interaction with HIS51 is also shown. (**C**) Docked Kushenol K in complex with NS3/NS2 protease (PDB ID: 2FOM); showing hydrogen bonds to the back-bone of PRO132, and to side chains of SER135 and TYR150. An arene interaction with HIS51 is also shown. LigX 2D interaction analysis shown at left and 3D interaction analysis shown at right side.

Likewise, in NS3/NS2B chimera Canthin-6-one 9-O-beta-glucopyranoside, Kushenol W and Kushenol K have been observed to bind through significant bonds with catalytic triad (His51-Asp75-Ser135) having binding score of −12.26, −11.68 and −11.30 kcal/mol, respectively. Side chains of all the essential catalytic residues (His51, Asp75 and Ser135), configuring the active site, were found to act as electron donor in forming a network of hydrogen bonds. Strong hydrophobic and non-covalent interactions were observed from other active site residues (Leu128, Phe130, Pro132, Tyr150, Gly151, Asn152, Gly153 and Tyr161) as detailed in Table 1B and illustrated in Fig. 5. The three ligands thus exerted hydrogen bonding interactions with the same catalytic triad residues, His51, Asp75 and Ser135 orienting in the same space in the catalytic site of the chimera. The residues implicated in hydrophobic and non-covalent binding were also found to be the same.

**Figure 6:**
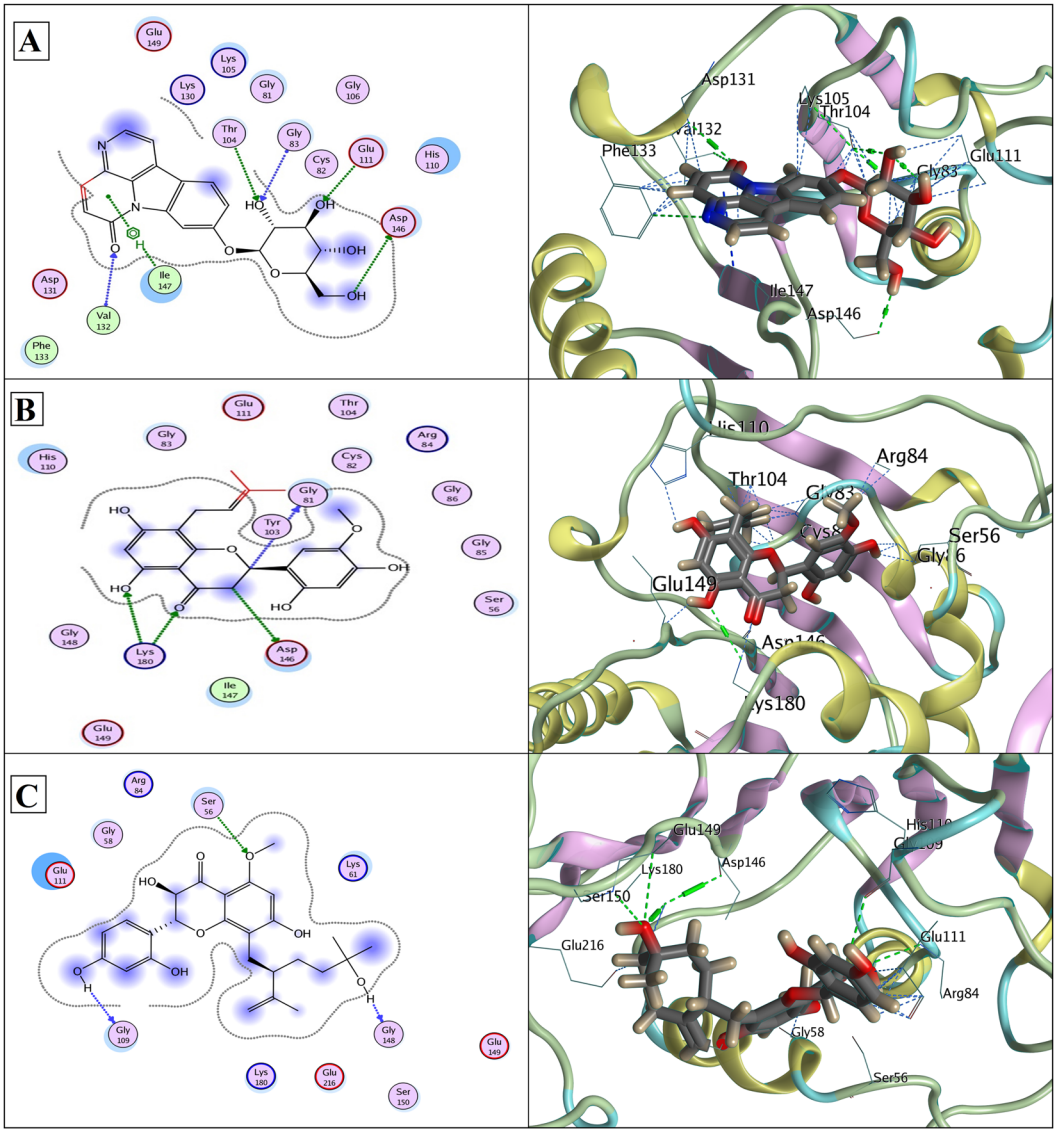
(**A**) Docked Canthin-6-one 9-O-beta-glucopyranoside in complex with NS5 RNA CAP (PDB ID: 3P97); showing hydrogen bonds to the back-bone of GLY83 and VAL132, and to side chains of THR104, GLU111 and ASP146. An arene interaction with ILE147 is also shown. (**B**) Docked Kushenol W in complex with NS5 RNA CAP (PDB ID: 3P97); showing hydrogen bonds to the back-bone of GLY81, and to side chains of ASP146 and LYS180. (**C**) Docked Kushenol K in complex with NS5 RNA CAP (PDB ID: 3P97); showing hydrogen bonds to the side chain of SER56, and back-bone of GLY109 and GLY148. LigX 2D interaction analysis shown at left and 3D interaction analysis shown at right side.

**Figure 7:**
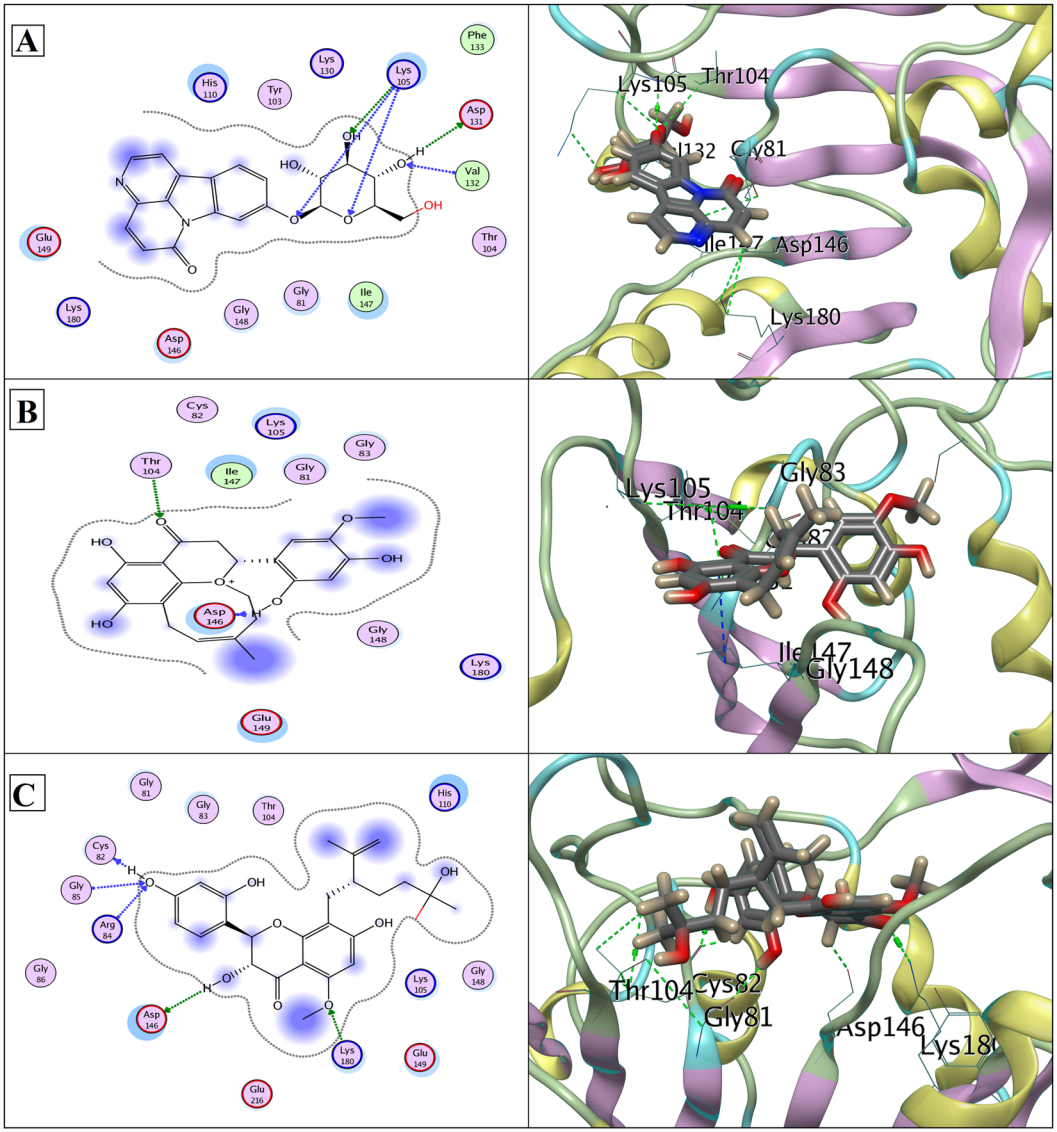
(**A**) Docked Canthin-6-one 9-O-beta-glucopyranoside in complex with NS5 SAM pocket (PDB ID: 3P97); showing hydrogen bonds to the side chain/back-bone of LYS105, to the side chain of ASP131, and to the back-bone of VAL132. (**B**) Docked Kushenol W in complex with NS5 SAM pocket (PDB ID: 3P97); showing hydrogen bonds to the side chain of THR104, and to the back-bone of ASP146. (**C**) Docked Kushenol K in complex with NS5 SAM pocket (PDB ID: 3P97); showing hydrogen bonds to back-bones of CYS82, GLY85 and ARG84, and to side chains of ASP146 and LYS180. LigX 2D interaction analysis shown at left and 3D interaction analysis shown at right side.

In case of NS5, phytochemical library was docked with the two functional domains; RNA pocket and SAM (*S*-adenosyl-_L_-methionine) pocket. In both the pockets of NS5, again Canthin-6-one 9-O-beta-glucopyranoside ranked at the top. In RNA pocket, the compound made hydrogen bonds to the back-bone of GLY83 and VAL132, and to side chains of THR104, GLU111 and ASP146 with a binding score −15.72. An arene interaction with ILE147 is also shown, as described in Table 1C1 and illustrated in Fig. 6A. The compound exhibited even stronger binding with NS5-SAM pocket, involving its three catalytic residues Asp131, Lys105, Val132 with a score of −14.53 as explained in Table 1C2 and Fig. 7A. Side chains and backbone atoms of these NS5 residues stabilized the inhibitor spatially within the pocket through hydrogen bonds. LigX interaction diagrams of Kushenol W/NS5-RNA pocket complex shows strong binding with Gly81, Asp146 and Lys180 through hydrogen bonds with a score of −14.55 as shown in Fig. 6B. Kushenol W was observed to form hydrogen bonds with side chain atoms of polar residue Thr104 and back-bone of acidic residue Asp146 in NS5-SAM pocket with a binding score of −13.91, Fig. 7B. Kushenol K showed hydrogen bonding with Ser56, Gly109 and Gly148 in NS5-RNA pocket with a binding score of −13.50, Fig. 6C. In Kushenol K/NS5-SAM complex, hydrogen bonding with Cys82, Arg84, Gly85, Asp146 and Lys180, with a binding score of −13.68 was observed, Fig. 7C. As mentioned above‚ these three inhibitors bound to common active site residues that are important structurally and functionally for the respective dengue proteases, Table 1C, Figs 6 and 7. However, for all the reported binding sites of the three NS proteins, Canthin-6-one 9-O-beta-glucopyranoside was ranked at the top as it exhibited maximum binding score and binding affinity.

In Figs 4–7, protein residues are shown as circles and their colouring represents their type. Residues with polar charge have mauve interior; however hydrophobic residues are shown as green circle. Acidic residues are further displayed with a red rim, and basic residues with a blue rim. Solvent exposure tempted by the ligands is shown as halo like disc in bluish colour around the residue. Ligands are shown according to residues types by default. Hydrogen bonds are shown with dotted lines arrows, representing the bond direction. The colour of interacting dotted lines arrow represents the location of residue in the protein, green for side chain residue, blue for backbone residue and yellow for solvent ion^32^. Detail list of LigX graphical keys with their description is given at Supplementary Fig. 5.

### ADMET/Drug scan results

ADMET based drug scan tool at Molinspiration server predicted the drug likeness of the proposed DV inhibitors. Canthin-6-one 9-O-beta-glucopyranoside (C_20_H_18_N_2_O_7_) is an alkaloid with a molecular weight of 398.37 g/mol, Log P value of 0.03; the compound contains four hydrogen bond donor (HBD) atoms and 8 hydrogen bond acceptor (HBA) atoms. Another flavonoid based phytochemical selected from the docked molecules is Kushenol W (C_21_H_22_O_7_) with molecular weight of 386.40 g/mol and LogP value of 3.82, it bears four HBD and seven HBA atoms. Kushenol K (C_26_H_32_O_8_) has a molecular weight of 72.53 g/mol, log P value of 3.87, with five HBD and eight HBA atoms. To further validate the inhibitors’ capability of drug likeliness, all the candidate molecules were subjected to ADMETsar server (Table 2). The ADMETsar analyses the compounds based on 4 parameters, absorption, distribution, metabolism, and excretion. These four parameters are then evaluated based on a number of thresholds. All the three inhibitors passed ADMETsar threshold of drug ability (Table 2).

**Table 2:** ADMET Profiling Enlisting Absoprtion, Metabloim And Toxicity related drug like parameters of Canthin-6-One 9-O-Beta-Glucopyranoside, Kushenol W and Kushenol K.

A. DMET Profiling			
Models	Canthin-6-one 9-O-beta-glucopyranoside	Kushenol W	Kushenol K
**A. Absorption**			
Blood-Brain Barrier	BBB+	BBB−	BBB−
Human Intestinal Absorption	HIA−	HIA +	HIA+
Caco-2 Permeability	Caco2−	Caco2+	Caco2+
P-glycoprotein Inhibitor	Non Inhibitor	Inhibitor	Inhibitor
Renal Organic Cation Transporter	Non Inhibitor	Non Inhibitor	Non Inhibitor
**B. Metabolism**			
CYP450 2C9 Substrate	Non Substrate	Non Substrate	Non Substrate
CYP450 2D6 Substrate	Non Substrate	Non Substrate	Non Substrate
CYP450 3A4 Substrate	Non Substrate	Substrate	Substrate
CYP450 1A2 Inhibitor	Inhibitor	Inhibitor	Non Inhibitor
CYP450 2C9 Inhibitor	Non Inhibitor	Inhibitor	Non Inhibitor
CYP450 2D6 Inhibitor	Non Inhibitor	Inhibitor	Non Inhibitor
CYP450 2C19 Inhibitor	Non Inhibitor	Inhibitor	Non Inhibitor
CYP450 3A4 Inhibitor	Non Inhibitor	Non Inhibitor	Inhibitor
AMES Toxicity	Non Ames toxic	Non Ames toxic	Non Ames toxic
**C. Toxicity**			
AMES Toxicity	Non Ames toxic	Non Ames toxic	Non Ames toxic
Carcinogens	Non carcinogens	Non carcinogens	Non carcinogens

### MD Simulation

**Figure 8:**
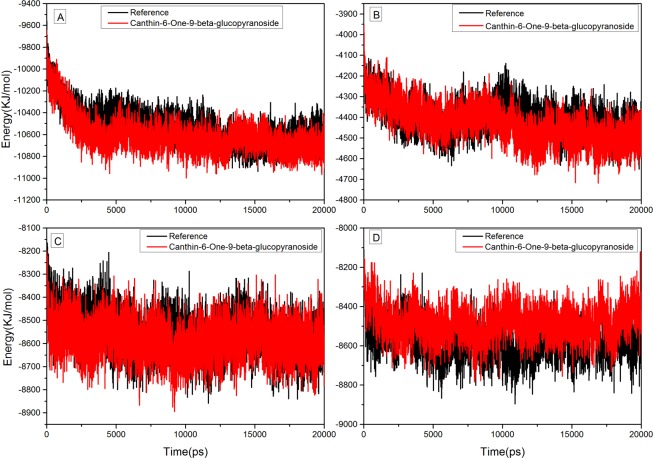
MD trajectories for NS1, NS3, NS5-RNA and NS5-SAM bound with and without Canthin-6-one 9-O-beta-glucopyranoside superposed over each other.

**Figure 9:**
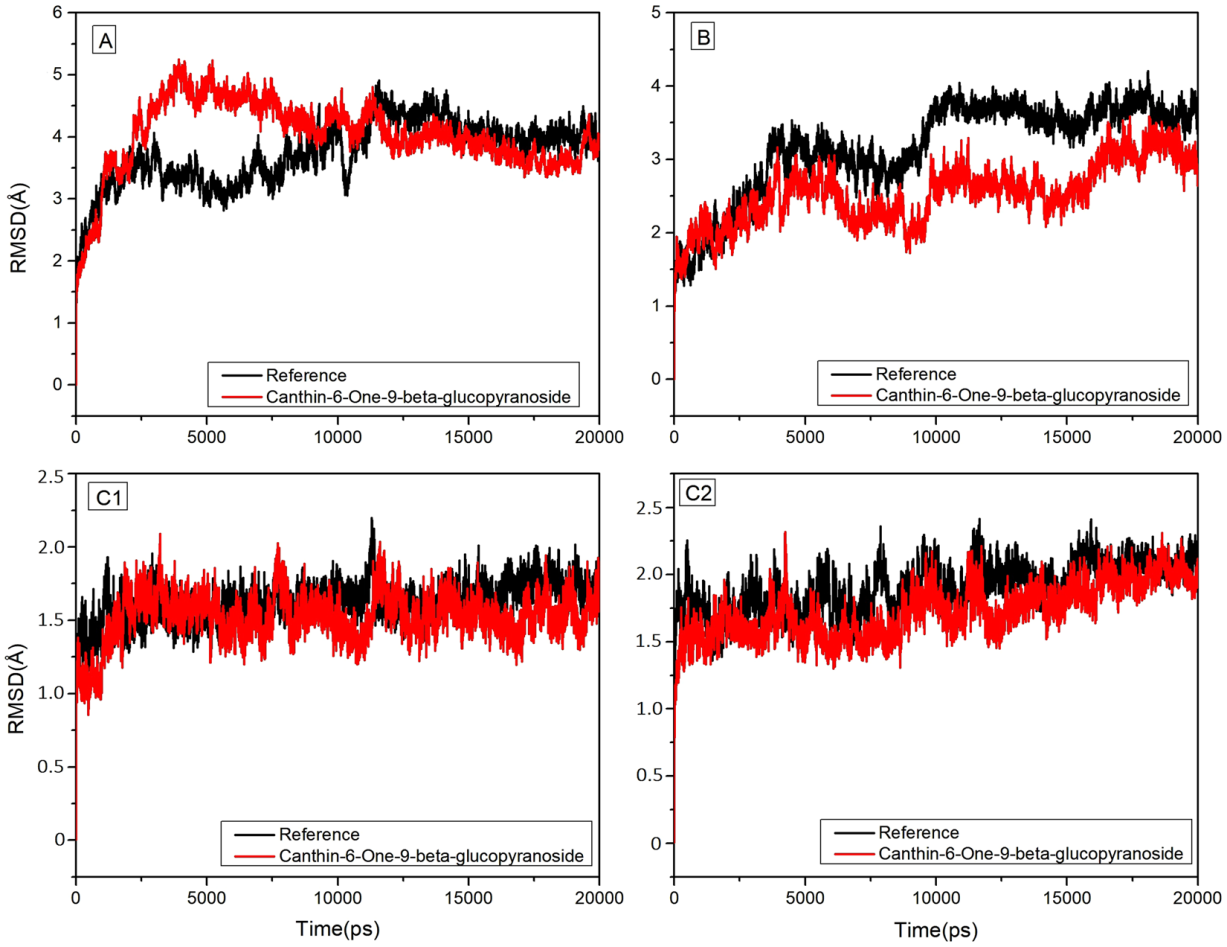
RMSD of Canthin-6-One 9-O-Beta-Glucopyranoside complexed to NS1 (**A**), NS3/N2B Chimera (**B**), NS5 RNA Pocket (C1) and SAM pocket (C2) with respect to their unbound (apo) reference structures.

As illustrated by docking studies, Canthin-6-one 9-O-beta-glucopyranoside showed strongest affinity with NS1, NS3/NS2B chimera, NS5-RNA and NS5-SAM pockets along with highest binding scores. To investigate the validity of the docking data and results, the docked complexes of Canthin-6-one 9-O-beta-glucopyranoside with NS1, NS3/NS2B chimera, NS5-RNA and NS5-SAM were MD simulated for 20 ns (Figs 8–9). MD trajectories generated by GROMACS for NS1, NS3, NS5-RNA and NS5-SAM bound with and without Canthin-6-one 9-O-beta-glucopyranoside superposed over each other are shown in Fig. 9. None of the complexes showed variation of total energy and distribution compared to that of native protein (Fig. 8). While in Fig. 9, binding of Canthin-6-one 9-O-beta-glucopyranoside to DV NS1, NS3, NS5-RNA and NS5-SAM is displayed as time series of the Cα atoms root-mean-square deviations (RMSD) bound with and without Canthin-6-one 9-O-beta-glucopyranoside. The relative fluctuation in the RMSD of Cα carbon atoms (Cα-RMSD) was observed to be the same for initial 3 ns of NS1 with and without ligand. However, RMSD for NS1 bound with Canthin-6-one 9-O-beta-glucopyranoside fluctuated more for 5–11 ns followed by a streak of continuous drop for the next 9 ns by the end of the simulation, representing convergence of the system. The overall RMSD ranged from 0 to 3.5 Å. Similarly, the RMSD fluctuation, in case of NS3/NS2 chimera bound with Canthin-6-one 9-O-beta-glucopyranoside, remained analogous to that of native DV NS3/NS2chimera onto 8 ns followed by a marginal decrease onto 16 ns converging in the end. However, no considerable variation of Cα-RMSD fluctuations between the ligand bound and native NS5-RNA and NS5-SAM proteins was observed, with exception that the ligand bound NS5-SAM showed a marginally higher fluctuation than that of native protein for the last 3 ns. Therefore, Canthin-6-one 9-O-beta-glucopyranoside showed a strong and stable interaction with all the 3 DV proteases over 20 ns simulation run except a little terminal fluctuation in case of the ligand bound NS5-SAM which needs to be explored over a longer MD run. The mean RMSD values are 1.9 Å, 1.4 Å, 0.86 Å and 1.5 Å for NS1 (Canthin-6-one 9-O-beta-glucopyranoside), NS3 (Canthin-6-one 9-O-beta-glucopyranoside) NS5/RNACAP (Canthin-6-one 9-O-beta-glucopyranoside) and NS5/SAM (Canthin-6-one 9-O-beta-glucopyranoside), respectively (Supplementary Table 1).

**Figure 10:**
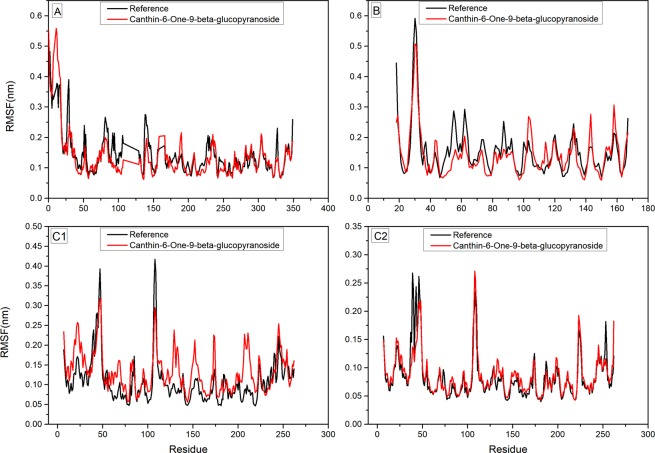
Root mean square fluctuations (RMSF) of Apo ((**A**)) NS1 (**B**) NS3 (C1) NS5/RNA and (C2) NS5/SAM compared to complexed states for 20 ns Simulation, Respectively.

Furthermore, Canthin-6-one 9-O-beta-glucopyranoside remained more stable throughout the simulations for NS5/SAM (RMSD mean = 1.52 Å, Max = 3.0 Å) and NS5/RNACAP (RMSD mean = 0.86 Å, Max = 1.7 Å). While NS1 and NS3/NS2B chimera took a longer time to reach equilibrium revealing that NS1 and NS3/NS2B chimera goes under rapid conformational changes. In case of NS5 (SAM) and NS5 (RNA) more number of intramolecular hydrogen bonds were observed throughout the simulations time. To better predict the raw conformational changes with respect to their secondary structures fluctuations we also calculated RMSF of our systems. Loops were quite flexible in NS1 and NS3/NS2B chimera during simulation. Fewer deviations with respect to the initial system indicate that ligand stayed inside the pocket. Root mean square fluctuation (RMSF) was calculated to compute atomic mobility of backbone atoms and high structural fluctuations over time. Calculated RMSF of each residue is given in Fig. 10. In case of NS1, larger local chain fluctuations were observed in N-terminus residues up to 0.5 nm. Binding site residues (Glu83 and Asn130) showed lesser fluctuations in bound state than apo-NS1. In the case of NS3- Canthin-6-one 9-O-beta-glucopyranoside complex, residues Gly29-Tyr32, Gly103, Lys104, Asn105, Lys143 and Ser158 tend to show more fluctuations as compared to critical binding pocket residues. Functionally important residues particularlyHis51 and Asp75 seemed more stable in complex as compared to its apo-NS3. In case of NS5 (RNA) and NS5 (SAM) that are majorly comprised of loop regions are susceptible to appreciable fluctuations. NS5 (RNA) showed considerable fluctuations in the complex form but the active site residues were more stabled in the complexed states as compared to the apo state. NS5 (SAM) showed more fluctuation than that of reference across residues 40–50 and 250–255 whereas the residues lying in the middle, 100–105 and 125–150, showed marginally higher fluctuation than that of the reference due to the fact that the binding site is configured by these residues. Overall, fluctuations were rather less in the complexed states as compared to the Apo state as overall system remained much stable as reflected by the corresponding RMSD trajectory. In addition to RMSF, the radius of gyration (R_g_) was also calculated to check the compactness of our systems understudy. R_g_ of all the systems was steady and consistent with RMSD and proteins remained stable and compact through the simulation run (Supplementary Fig. 6). Aforementioned MDS analysis highlights that ligand protein complexes were overall stable saving a few fluctuations. The variation in the individual trajectories was observed due to the fact that the target active site residues were different in each of the case although the ligand was same. However, the behavior of binding as illustrated by docking and MDS results fairly demonstrates that the ligand would serve as a potent inhibitor for the DV proteins.

## Discussion

Nonetheless of present upsurge in efforts for discovery of vital anti-dengue prophylactic and curative drugs, no FDA approved therapeutic and/or preventive regime has yet reached to the market. By the end of 20^th^ century *in vitro* study reported the simultaneous inhibitory effect of Ribavirin against DV 1–4 but *in vivo* testing on mice reported minimal inhibitory effect of ribavirin alone and various conjugated therapies were recommended to achieve prophylactic ribavirin treatment^33,34^. To offer long lasting immunization against dengue infections, Sanofi and Pasteur Institute jointly launched four anti dengue vaccines CYD-TDV, CYD1, CYD2, CYD3 and CYD4, but clinical studies couldn’t produce reliable cross protection against all DV serotypes^35^. A new tetravalent vaccine TV003 that acts to boost immune response against heterotypic re-infections has reached to phase 3 of clinical evaluation with satisfactory safety profile^36^. Alternatively, monoclonal antibodies that are designed specifically against individual DV structural proteins i.e. precursor membrane (preM) and envelop (E) protein are newly recommended class of vaccines for immune response enhancement during dengue infection. Recently, an antibody mAb2D22 has been proved to be specific for DV2 immature viruses but couldn’t generate immune response for mature DV^37^. Till now none of the synthetic medicine has enough clinical evidence to prove its efficacy against all DV serotypes.

Owing to the phenomenon of Antibody-Dependent Enhancement (ADE) in DV infection, neutralizing all genotypes within all DV serotypes by targeting their quaternary structures is a challenging task but over the time this idea gained acceptance. A study by Li *et al*. (2005) supported and emphasized on this concept by revealing >60% protein sequence identity in NS3 proteases of all the four DV serotypes and high functional conservation in NS3 substrate binding pocket within all DV serotypes functional profiling analysis. Furthermore, they concluded that it was possible to develop a single inhibitor which could target NS3 protease of all DV serotypes^26^. Kinney *et al*. 2005 claimed the reduction in viremia titer of all DV serotypes by the treatment of 10 µM arginine rich peptide, 3′CS to very low detectable level. They clearly demonstrated the anti-dengue potential of this compound by inhibition of viral replication in Vero cell cultures^29^. In 2010, Chandramouli and his coworkers revealed an innovative and surprising structural similarity in overall fold of NS3 protease core and warranted more research on targeting NS3 for pan-serotype based anti-dengue drug development^38^. Small benzimidazole derivative, MB21 was proposed as a single drug like molecule that would inhibit the activity of DV NS2 protein of all the DV serotypes^39^. Recently, Mirza *et al*. (2017) reported four potent chemical compounds for successful simultaneous *in silico* inhibition of NS3 proteases of four DV serotypes^10^.

None of the synthetic medicine has enough clinical evidence to prove its efficacy against all DV serotypes. Phytochemicals usage as antiviral drug opens new window for researchers over the past few years. Several studies reported handful numbers of antiviral phytochemical entities against individual dengue viral structural/non-structural proteases specifically against NS2B–NS3 protease^12,40,41^. Likewise, Mir *et al*. (2016) proposed quercetin, a flavonoid based natural extract of *Carica papaya* that has potential to disrupt the structural integrity of envelope E protein and halts the fusion process and binding of dengue virus to host surface^19^. Ruchi and his coworkers^27^ recently screened an antiviral drug from Indian herb *Cissampelos pareira* Linn. Proposed phytochemical established broad protection of this phytochemical against all DV serotypes through cell based assays and mouse model testing but this drug has not yet reached to preclinical development phase. *In vitro* and *in vivo* studies are evident that extract of *Cissampelos pareira* Linn acts as a potent downregulatory factor of pro-inflammatory signaling and has the potential to lower viremia during infection^27^.

In this study, we screened novel cost-effective multi-target drug like molecules, of plant origin and with desired ADME-Tox characteristics by exploiting conserved functional regions of DV proteins. Aiming that goal, *in silico* structure based drug design approach was designed to disrupt viral replication, host-viral interaction and infectivity cycle by screening inhibitors against NS1, NS3 and NS5 non-structural proteins. Three potent common antiviral inhibitors including Canthin-6-one 9-O-beta-glucopyranoside, Kushenol W and Kushenol K were screened based on their binding affinity and score. Asn130 has been found crucial for, N-glycosylation, stability and interactions of NS1 with other proteins and its mutation has been reported to decrease secretion and viral replication^8,9,41^. Asn130 in addition to Asn175 and Asn207 constitute three N-linked glycosylation sites in NS1 and enhance pathogenicity by contributing to viral replication^8,9^. Disruptions of N-glycosylation sites of NS1 protein result in hexamer disability and poor interactions with host synthetic machinery. Deoxycalyxin, a flavonoid based drug like molecule has been reported to bind Asn130 and impairs NS1 ability to replicate. Blocking two N-linked glycosylation sites (Asn76 & Asn130) would compromise the glycosylation, thereby inhibiting the viral activity within the host cell^41^. NS3/NS2B protease has been a target in various drug discovery investigations because of its central role in enhancing viral replication in the host cells. Li *et al*.^26^ reported Gln-35, Leu-128, Asn-152, Pro-132 and Val-155 as completely conserved residues of NS3/NSS2B substrate binding pockets. All the completely conserved residues reported in this study are shown to bind our three drug leads in Fig. 5. Within the NS3 protease Tyr-150 is known to stabilize the substrate through π interaction. Tyr-150 is also evident in stabilizing our proposed inhibitors within the substrate binding pocket of NS3 protease^26^. Crucial catalytic triad residues specifically His51, Asp75 and Ser135, if inactivated render pathogenicity, diminished and/or compromised^10,11,23,42^.

Our proposed drug like molecules are found to make strong H-bonds in addition to hydrophobic interactions with already known crucial active residues of DV proteins, disrupting their functions crucial for viral replication, thereby inhibiting DV infectivity. Research has already come up with potential inhibitors against DV proteins through both, the structure as well as ligand based, screening methods. Lim and his coworkers^16^ proposed potent druggable compounds capable to inhibit NS5 protein through structure-based and ligand-based virtual screening. Whilst, inhibitors screened in the current study show more strong binding and significant inhibition potential against NS1, NS3/NS2B chimera, NS5-RNA and NS5-SAM pockets with optimum binding affinity. Subtle interactions of essential catalytic residues of NS1, NS3 and NS5 proteins with three proposed phytochemical inhibitors are given in Figs 4–7. MD simulation trajectories showed that there were little trails of cα-RMSD variations in ligand bound DV inhibitors compared to unbound DV proteins in the beginning, but the systems turned to normal in the end of the simulation runs with lower average free energy. This validates that interactions predicted by docking were stable and stronger.

Natural source of Canthin-6-one 9-O-beta-glucopyranoside is *Eurycoma harmandiana*, which is a small plant belonging to genus *Eurycoma Jack* of the *Simaroubaceae* family and distributed in Asia^43^. While, natural source of Kushenol W and Kushenol K is *Sophora flavescens. Sophora flavescens* is a Chinese medicinal herb of *Sophora* genus, a genus of the *Fabaceae* family, and widely distributed in Asian regions^44^. Canthin-6-one 9-O-beta-glucopyranosideas has already been reported for unique properties to stabilize human red blood cell membrane and with no known cytotoxic effect; it has been proposed as a key NF-κB inhibitor in cancer cell line^45^. Apart from its anti-inflammatory effect, this compound has also been shown to possess antiulcer, antimalarial and anti-plasmodial properties^45^. Similarly, Kushenol K has also been reported to be a potent anticancer agent, inhibiting estrogen α-receptor and thus eventually reducing over expression of estrogen in breast cancer^46^. While role of Kushenol W has not yet been investigated in any scientific study.

Our effort of targeting all known DV serotypes simultaneously by exploiting their structural and functional conservation yielded promising results. This study identified three inhibitors with strong potential of drug leads capable to inhibit all the four serotypes of the DV proteins. The set of compounds identified in this study could possibly function synergistically or additively against all dengue serotypes. This is particularly important against viruses which keep evolving continuously due to higher rate of mutation. The benefit of treatment strategies involving synergism has been already reported in case of HIV and HCV infections^47,48^. These inhibitors may lead to one therapeutic solution against the diversity of DV serotypes by efficiently targeting and inhibiting the catalytic function of three functionally conserved non-structural proteins. Therefore, our findings regarding bioactivity of Canthin-6-one 9-O-beta-glucopyranoside, Kushenol W and Kushenol K warrant further experimental work for structure based leads optimization.

## Materials and Methods

### Analysis for conserveness among DV serotypes

An iterative and exhaustive multiple sequence analysis was carried out to find out the evolutionary conserved functional regions among four dengue serotypes which could be further used as potent targets for the discovery of a drug lead which could universally inhibit all the DV serotypes. Firstly, full length genome sequences of all the four serotypes including DV1 (NC_001477.1), DV (NC_001474.2), DV3 (NC_001475.2) and DV4 (NC_002640.1) were retrieved from NCBI, the genomes were aligned using Mega v6.0^49^. For visual representation of aligned genomic data of four DV serotypes, Circos plot was generated through Circos tool by taking DV serotype 2 as reference genome^50^. DV serotype 2 was taken as reference as it is considered the most virulent strain of all 4 serotypes^51^. Genome analysis of DV serotypes for sequence identity, similarity, variation of GC content and other genetic features was done through multiple Perl scripts and resultant configuration files were used to communicate this information through Circos plot^50^.

High sequence identity and homology within DV serotypes in NS1, NS3/NS2B chimera and NS5 non-structural proteins at genome level was also validated at protein sequence and structure level. Well-conserved protein motif localized within NS1, NS3/NS2B and NS5 of four DV serotypes were analyzed through Clustal Omega multiple sequence alignment of amino sequences taken from UNIPROT^49,52^. To ensure broad spectrum relevance of these protein targets, conserved functional motif recognition within active pockets were analyzed through structural alignment of four DV serotypes^49,53^. For superposition, we retrieved 3D structures of the different DV serotypes NS proteases from RCSB PDB. As the structures of some DV serotypes NS proteins have not been determined yet, thus we predicted their 3D structures using homology modeling in Chimera v1.11.2^54^. Detail about PDB retrieved and computational predicted structures is enlisted in supplementary Table 2.

### Selection and refinement of DV receptor proteins

Solved structures of DV NS1, NS3 and NS5 proteins of reference DV-2 serotype were taken from Protein Data Bank (PDB) with PDB ID’s 4O6B^8^, 2FOM^55^ and 3P97^16^, respectively. Retrieved structures were prepared for docking through Molecular Operating Environment (MOE).

### Ligand database preparation

An intensive literature search was performed to search the phytochemicals reported for activity against flaviviruses. Chemical structures of phytochemicals were taken from MPD3 database^24^, MAPS database^56^, Pubchem^57^ and Zinc database^58^ in multiple ligand file formats i.e. sdf, mol, mol2. Ligand optimization of all these ligands structures was done in MOE by adding partial charges using Protonate3D module. Energy minimization of each ligand was done by using MMFF94X force field. Afterwards each of the selected ligands was added individually to the MOE ligand database for docking purpose.

### Molecular docking

Search for already reported potential binding residues of DV NS1, NS3 and NS5 proteins was done through MOE site finder tool and electrostatic surface map was generated around them to describe the docking site. MOE Dock tool was used to dock a ligand database of 10,326 phytochemicals within the defined docking sites of NS1, NS3/NS2B and NS5 proteins. Triangular matcher algorithm was applied as default ligand placement methods to find 1000 best poses of docked molecules^59^.

Rescoring of simulated poses was done by London dG scoring function. Top 10 ranked poses per molecule generated by London dG were further minimized by Force field refinement algorithm in which final binding energy is calculated through Generalized Born solvation model while keeping receptor residues rigid. All the compounds were ranked based on S-score, binding affinity and Root-Mean-Square Deviation (RMSD) values. From the top ranked poses, idea was to pick only those compounds for further analysis that bind to active residues of dengue proteins with favorable dock score. Already reported co-crystallized inhibitors were redocked as reference ligands with NS1, NS3/NS2B and NS5 proteins using MOE to validate docking protocol employed to predict the binding orientations of phytochemicals within the catalytic pockets.

### Ligand receptor interaction analysis

For a clear view of receptor ligand interaction of the best docked complexes, 2D plots of receptor ligand interactions were analysed through LigX tool of MOE. It generates a 2D graph of electrostatic interactions, hydrogen bonding, Van der Waals forces and hydrophobic interactions which contribute to the affinity of drug like molecules within the active site of DV NS proteins. 3D images of DV protein inhibitor complexes were generated through MOE^60^.

### ADME Toxicity/Drug scan

Computational approximation of the docked phytochemical’s drug likeliness was found on the basis of thresholds set by “Lipinski’s Rule of Five” through Drug scan tool at Molinspiration server^61,62^. The qualitative assessment of absorption, deposition, metabolism, excretion and toxicity profile of these hits were predicted virtually by using ADMETsar server^63^. In addition to this, AMES Toxicity and carcinogenic likelihood of inhibitors was also evaluated^64^.

### Molecular dynamics and simulations

Out of best docked complexes those with Canthin-6-one 9-O-beta-glucopyranoside inhibitor were subjected to Molecular Dynamics (MD) simulations and free energy calculations as the ligand exhibited strong binding affinity, illustrated by high dock score and favorable molecular interaction network. For comparison and control apo structures of NS1, NS3/NS2B Chimera, NS5 RNA pocket and NS5 SAM pocket, and corresponding Canthin-6-one 9-O-beta-glucopyranoside bound complexes were subjected to MD Simulation. Explicit solvent MD simulations were conducted using GROMACS v5.1.4 and ligand topology files were built using CHARMM force field through CGenFF server^64–66^. Ligand-protein complexes were solvated in octahedron box with TIP3P water model. Particle Mesh Ewald was used to calculate Long- range electrostatics. For short range van-der Waals and electrostatics, a cut-off value of 10 Å was employed^67^. System temperature was stabilized gradually from 0k to 300 k for 50 ps under NVT ensemble. Furthermore, the system was simulated under NPT ensemble at 300 K temperature and pressure 1.0 bar^68^. Linear Constraint Solver (LINCS) algorithm was employed for all bonds constraints^69^. Finally, 20 ns production run was carried out and coordinates of the system were saved after every 2.0 fs for post processing analysis.

## Supplementary information

Supplementary Information
