# Supplementary material for: Computational screening of medicinal plant phytochemicals to discover potent pan-serotype inhibitors against dengue virus
Source: Sci Rep. 2019 Feb 5;9:1433. doi: 10.1038/s41598-018-38450-1 (PMC6363786; doi:10.1038/s41598-018-38450-1)
Supplement: Supplementary file 1 — Supplementary Information [file 41598_2018_38450_MOESM1_ESM.docx]

**Computational screening of medicinal plant phytochemicals to discover potent pan-serotype inhibitors against dengue virus**

**Muhammad Tahir ul Qamar^1,Ϯ,*^, Arooma Maryam^2,Ϯ^, Iqra Muneer^3,Ϯ^, Feng Xing^1^, Usman Ali Ashfaq^4^, Faheem Ahmed Khan^5^, Farooq Anwar^6^, Mohammed H. Geesi^7,*^, Rana Rehan Khalid^2^, Sadaf Abdul Rauf^8^, Abdul Rauf Siddiqi^2,*^**

^1^College of Informatics, Huazhong Agricultural University, Wuhan, P.R. China

^2^Department of Biosciences, COMSATS University Islamabad (CUI), Islamabad, Pakistan

^3^School of Life Sciences, University of Science and Technology of China, P.R. China

^4^Department of Bioinformatics and Biotechnology, Government College University Faisalabad, Faisalabad, Pakistan

^4^Key Laboratory of Agricultural Animal Genetics, Breeding and Reproduction, Ministry of Education China, Huazhong Agricultural University, Wuhan, P.R. China

^6^Department of Chemistry, University of Sargodha, Sargodha, Pakistan

^7^Department of Chemistry, Faculty of Sciences and Humanities, Prince Sattam Bin Abdulaziz University, Al Kharj, Saudi Arabia

^8^Department of Computer Science, Fatima Jinnah Women University, Rawalpindi, Pakistan

**^Ϯ^**These authors contributed equally in this work

***Correspondence authors:**

Muhammad Tahir ul Qamar ([m.tahirulqamar@webmail.hzau.edu.cn](mailto:m.tahirulqamar@webmail.hzau.edu.cn));

Mohammed H. Geesi ([m.geesi@psau.edu.sa](mailto:m.geesi@psau.edu.sa));

Abdul Rauf Siddiqi ([abdulraufsher@gmail.com](mailto:abdulraufsher@gmail.com))

# Supplementary Tables

**Supplementary Table 1:** Average and maximum RMSD values of Canthin-6-One 9-O-Beta-Glucopyranoside in complex with NS1, NS3/NS2B Chimera, NS5 RNA Pocket and SAM pocket relative to apo (reference) structure.

| Complex | Average (RMSD Å) | Max (RMSD Å) |
| --- | --- | --- |
| NS1 (Reference) | 1.93 | 3.86 |
| NS1 (Canthin-6-one 9-O-beta-glucopyranoside) | 1.96 | 3.91 |
| NS3(Reference) | 1.64 | 3.28 |
| NS3 (Canthin-6-one 9-O-beta-glucopyranoside) | 1.37 | 2.74 |
| NS5/RNACAP(Reference) | 0.81 | 1.63 |
| NS5/RNACAP (Canthin-6-one 9-O-beta-glucopyranoside) | 0.86 | 1.72 |
| NS5/SAM(Reference) | 1.00 | 2.00 |
| NS5/SAM (Canthin-6-one 9-O-beta-glucopyranoside) | 1.52 | 3.00 |

**Supplementary Table 2:** Detail of PDB retrieved and computational predicted structures used in superposition analyses.

| DV serotypes | NS proteins | Homology model predicted | PDB retrieved model | PDB IDs |
| --- | --- | --- | --- | --- |
| DV-1 | NS1 | - | Yes | 4OIG |
|  | NS3/NS2B | - | Yes | 3L6P |
|  | NS5 | Yes | - | - |
| DV-2 | NS1 | - | Yes | 4O6B |
|  | NS3/NS2B | - | Yes | 2FOM |
|  | NS5 | - | Yes | 3P97 |
| DV-3 | NS1 | Yes | - | - |
|  | NS3/NS2B | - | Yes | 3U1J |
|  | NS5 | - | Yes | 4V0Q |
| DV-4 | NS1 | Yes | - | - |
|  | NS3/NS2B | - | Yes | 2JLR |
|  | NS5 | Yes | - | - |

# Supplementary Figures


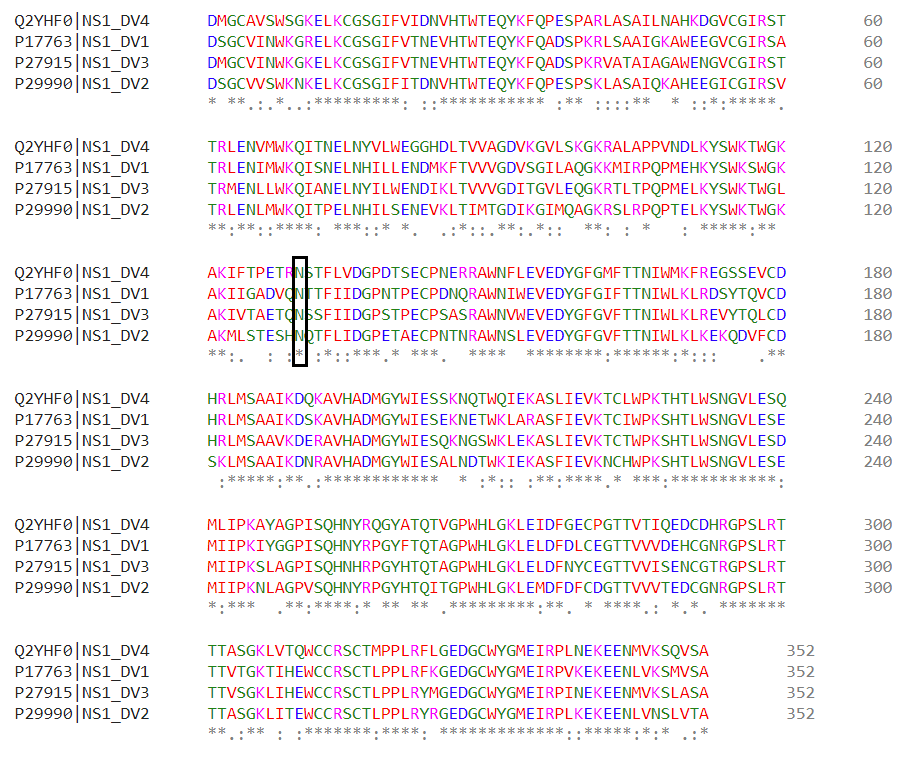


**Supplementary Figure 1:** Multiple sequence alignment of NS1 protein from DV serotypes 1-4; target conserved residue Asn130 shown in black box.


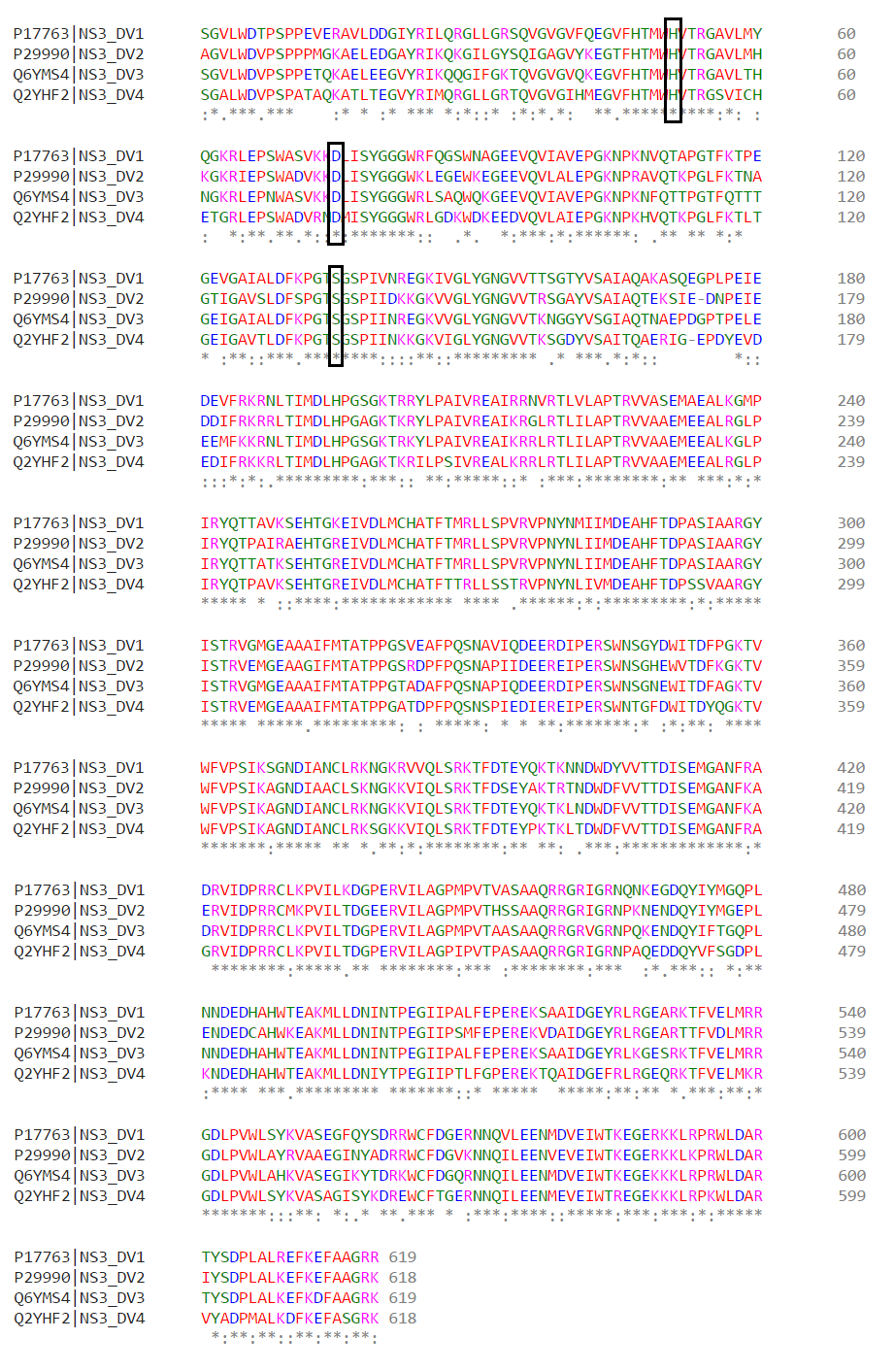


**Supplementary Figure 2:** Multiple sequence alignment of NS3/NS2B protease from DV serotypes 1-4; target conserved residues His51, Asp75, Ser135 shown in black boxes


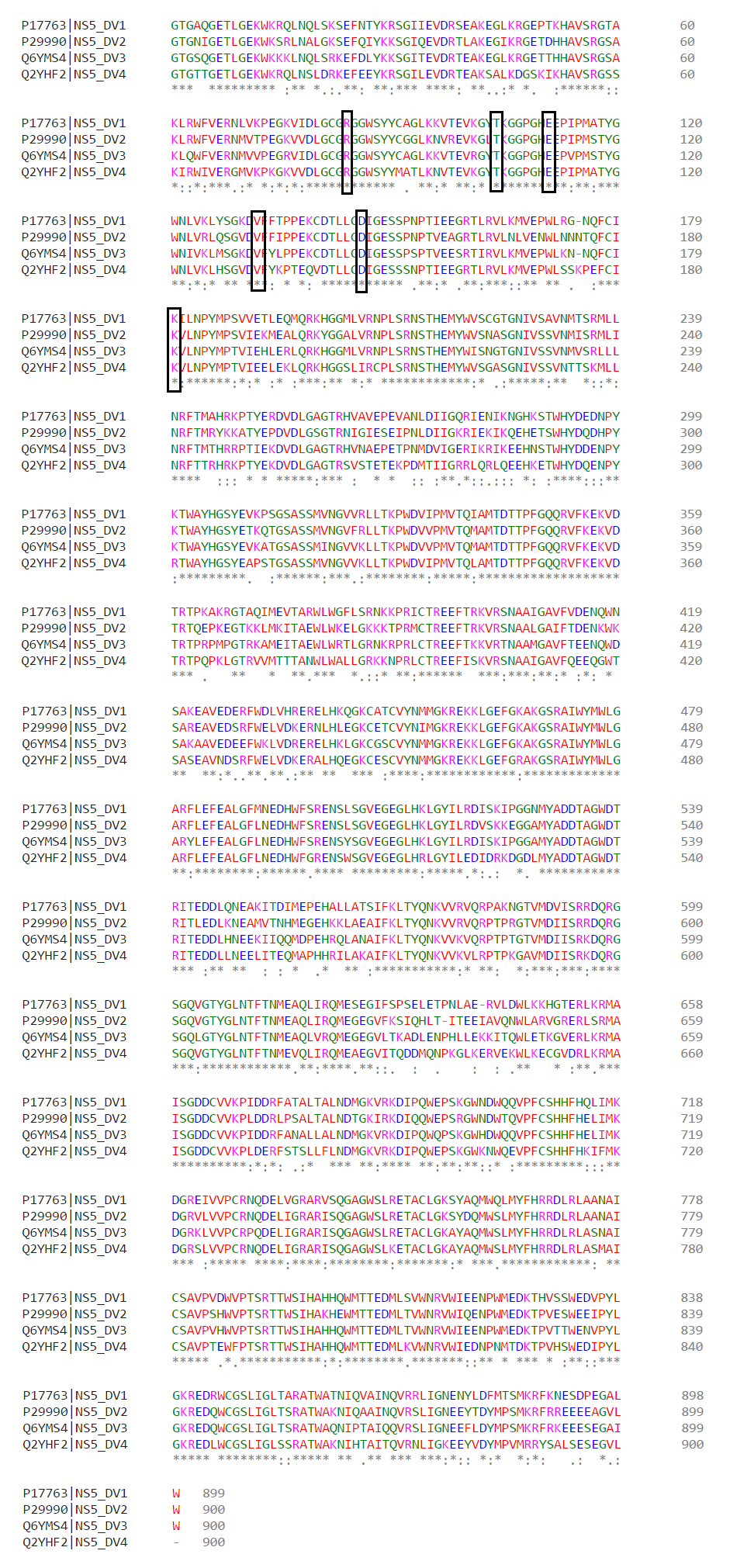


**Supplementary Figure 3:** Multiple sequence alignment of NS5 protein from DV serotypes 1-4; target conserved residues Arg84, Thr104, Glu111, Val132, Asp146, Lys180 shown in black boxes


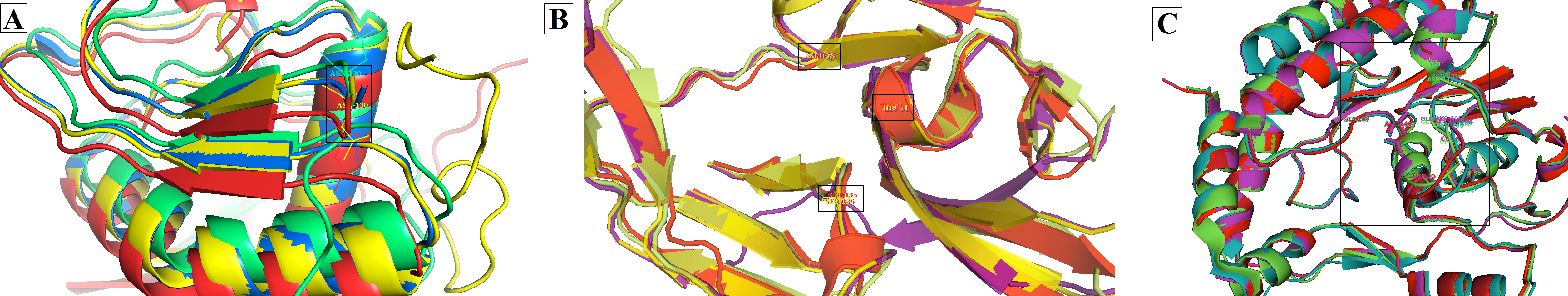


**Supplementary Figure 4:** 3D Structural Superimposition of NS1, NS3/NS2B and NS5 proteins from DV serotypes 1-4 (A, B, C respectively); target residues are found conserved throughout the binding pockets of respective proteins and shown in black boxes


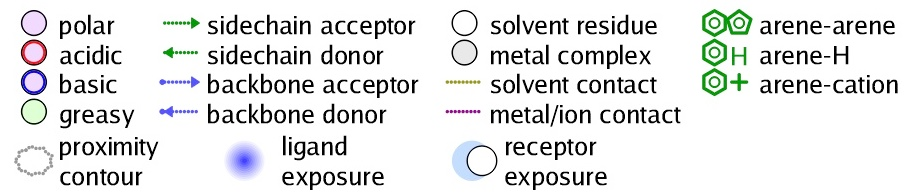


**Supplementary Figure 5:** Detail description of LigX keys


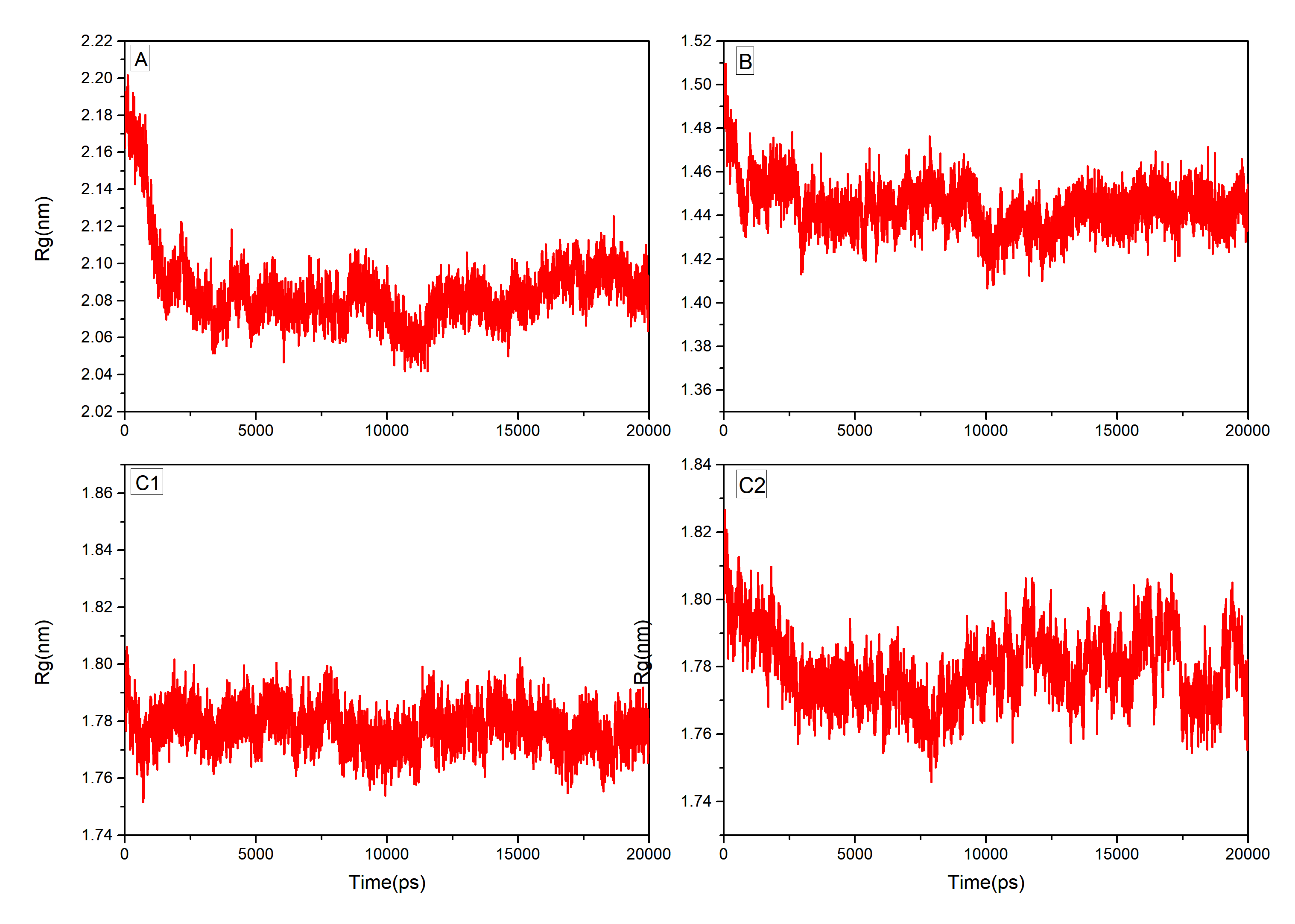


**Supplementary Figure 6:** Radius of Gyration (Rg) of all the complexed systems for 20ns MD simulation (A) NS1 (B) NS3 (C1) NS5/RNA and (C2) NS5/SAM, Respectively.
